# Magnitude of Multidrug Resistance among Bacterial Isolates from Surgical Site Infections in Two National Referral Hospitals in Asmara, Eritrea

**DOI:** 10.1155/2021/6690222

**Published:** 2021-02-26

**Authors:** Eyob Yohannes Garoy, Yacob Berhane Gebreab, Oliver Okoth Achila, Nobiel Tecklebrhan, Hermon Michael Tsegai, Alex Zecarias Hailu, Abrehet Marikos Buthuamlak, Tewelde Ghide Asfaga, Mohammed Elfatih Hamida

**Affiliations:** ^1^Department of Clinical Laboratory Sciences, Orotta College of Medicine and Health Sciences (OCMHS), Asmara, Eritrea; ^2^Department of Clinical Laboratory Sciences, Asmara College of Health Science (ACHS), Asmara, Eritrea

## Abstract

**Background:**

The World Health Organization has emphasized the importance of understanding the epidemiology of MDR organisms from a local standpoint. Here, we report on a spectrum of bacteria associated with surgical site infections in two referral hospitals in Eritrea and the associated antibiotic susceptibility patterns.

**Methods:**

This survey was conducted between February and May 2017. A total of 83 patients receiving treatment for various surgical conditions were included. Swabs from infected surgical sites were collected using Levine technique and processed using standard microbiological procedures. *In vitro* antimicrobial susceptibility testing was performed on Mueller–Hinton Agar by the Kirby-Bauer disk diffusion method following Clinical and Laboratory Standards Institute guidelines. The data were analyzed using SPSS version 20.

**Results:**

A total of 116 isolates were recovered from 83 patients. In total, 67 (58%) and 49 (42%) of the isolates were Gram-positive and Gram-negative bacteria, respectively. The most common isolates included *Citrobacter* spp., *Klebsiella* spp., *Escherichia coli*, *Proteus* spp*., Pseudomonas aeruginosa, Salmonella* spp., *Enterobacter* spp., and *Acinetobacter* spp. In contrast, *Staphylococcus aureus,* CONS, and *Streptococcus viridians* were the predominant Gram-positive isolates. All the *Staphylococcus aureus* isolates were resistant to penicillin. MRSA phenotype was observed in 70% of the isolates. Vancomycin, clindamycin, and erythromycin resistance were observed in 60%, 25%, and 25% of the isolates, respectively. Furthermore, a high proportion (91%) of the Gram-negative bacteria were resistant to ampicillin and 100% of the *Pseudomonas aeruginosa* and *Escherichia coli* isolates were resistant to >5 of the tested antibiotics. The two *Acinetobacter* isolates were resistant to >7 antimicrobial agents. We also noted that 4 (60%) of the *Klebsiella* isolates were resistant to >5 antimicrobial agents. Possible pan-drug-resistant (PDR) strains were also isolated.

**Conclusion:**

Due to the high frequency of MDR isolates reported in this study, the development and implementation of suitable infection control policies and guidelines is imperative.

## 1. Background

Surgical site infections (SSIs), a subcategory of nosocomial infections or healthcare-associated infections, are regarded as an important adverse postoperative event [1]. In terms of frequency and costs, a report from the USA indicated that SSIs are second in frequency and third in cost [1]. The predominant bacterial isolates in hospital-acquired SSIs include *Staphylococcus aureus, Enterococcus* spp., *Citrobacter* spp*., Pseudomonas aeruginosa*, *Klebsiella pneumoniae*, *Escherichia coli*, and other *Enterobacteriaceae. Acinetobacter baumannii* is another bacterium that is causing increasing concern in different hospital departments (internal medicine, intensive care units (ICUs), surgery) and long-term-care settings [2, 3].

Importantly, molecular profiles of isolates from apparently unrelated infections have reported widespread interspecies transfer of genes encoding antibiotic resistance, adding another facet to the threat posed by these organisms [1]. In fact, a high level of resistance to the commonly prescribed antibiotics like penicillin, ampicillin, tetracyclines, trimethoprim-sulfamethoxazole (TMP-SMX), chloramphenicol, and third-generation cephalosporins (cefoxitin, cefotaxime, and ceftriaxone) has been described in multiple countries in sub-Saharan Africa (SSA) [4–7].

In general, multidrug resistance (MDR) (concomitant resistance to ≥3 different antimicrobial classes) is a common trait in these isolates [1, 6, 8, 9]. In particular, extended spectrum-*β*-lactamases (ESBLs, class A) and AmpC *β*-lactamases (class C)-producing *Escherichia coli* and *Klebsiella species* have been described [6]. Previous reports of isolates that are resistant to second-generation cephalosporins and *β*-lactam/*β*-lactamase inhibitor combinations in the absence of ESBL-containing plasmids exist [10]. In addition, healthcare-associated methicillin-resistant *Staphylococcus aureus* (HA-MRSA) oxacillin/nafcillin-resistant *Staphylococcus aureus* (ORSA); penicillin-resistant *Streptococcus pneumoniae* (PRSP); vancomycin-resistant enterococci (VRE), and carbapenem-resistant and fluoroquinolone-resistant *Pseudomonas aeruginosa* and *Enterobacteriaceae* have also emerged as important families of healthcare-associated pathogens worldwide [6, 11]. Overall, multidrug-resistant (MDR) isolates complicate treatment and often result in extended hospital stay and higher morbidity and mortality [1].

At present, the emergence and rapid global spread of MDR threatens the gains made in the previous decades on the treatment and control of multiple bacterial infections. Importantly, multiple reports have consistently demonstrated that the frequency of MDR-associated SSI has increased globally over the last decade [1, 6, 12] with low- and medium-income countries (LMICs) reporting comparatively higher frequencies [8, 11, 13]. In Eritrea, a recent multicenter study noted that the frequency of HA-MRSA in hospitalized patients with wound infections was 72% [14]. Altogether, existing data suggest a highly heterogeneous picture with significant intra- and intercountry variation potentially reflecting the multiplicity of infection control practices (or lack thereof) and antimicrobial prescription patterns within the region [1, 9].

Although the significance of gathering accurate data on resistance to antibacterial agents is well recognized, the problem is largely underinvestigated and/or underreported in LMICs in SSA. For instance, obtaining adequate country-specific data on multiple facets of SSI is difficult despite the rapid expansion in facilities offering complex surgical procedures in urban and rural settings in SSA. And where data exists, it is often suboptimal/partial or conflicting. In Eritrea, data on the antimicrobial profiles of GNB or SSIs are lacking in international literature. In this regard, the true caseload of hospital-acquired SSIs in Eritrea is largely unknown. Adding to this concerning phenomenon is the fact that existing infection control practices in the country are largely rudimentary. To illustrate, the country has no infrastructure of trained infection-control professionals and lacks a laboratory-based surveillance and reporting system. Internment of multiple patients (often in close proximity) in single wards also limits the utility of contact precaution procedures. Moreover, antimicrobial therapy is infrequently guided by laboratory-generated drug susceptibility results or antibiotic stewardship programs.

Therefore, this study was designed to collect data on the spectrum of bacteria associated with SSIs in two referral hospitals in Eritrea. Phenotypic antibiotic resistance patterns were also profiled. Understanding the overall epidemiology of SSI from a local standpoint may be useful in guiding SSIs therapy and infection-control interventions such as screening and contact precautions, among others. The study may also provide crucial data on the magnitude of the problem in the country.

## 2. Methods

### 2.1. Study Design and Settings

This survey was conducted between February and May 2017 in two referral hospitals (Halibet National Referral Hospital and Orotta National Referral Hospital) in Asmara, Eritrea.

### 2.2. Study Population and Patient Recruitment

Male and female patients admitted in the hospital surgical wards after surgery were included in the study. Mostly, they were patients who developed infection after 48 hours of admission. Common indications included amputations, colonoscopies, appendectomies, and fixations associated with compound fractures and surgical debridement,among others. Pediatric patients, receiving treatment for various surgical conditions (and meeting the pre-set criteria), were also included. In this situation, consent to participate in the study was obtained from the parents.

### 2.3. Sample Collection and Processing

The infected sites (wound bed) were prepared prior to specimen collection by using Levine's technique [15]. The technique outlines the appropriate wound preparation procedures that should precede sample collection. To collect samples, a sterile cotton-tipped applicator was rotated gently over 1 cm^2^ area for 5 seconds. In the process, the pressure applied was sufficient to express the purulent exudates. Double wound swabs were subsequently taken from each wound.

### 2.4. Laboratory Procedures

#### 2.4.1. Identification of Isolates

Specimens (wound swabs) were processed at the National Health Laboratory Bacteriology Laboratory within 1 hour after collection. One swab was used to make Gram stain smears. The other swab was inoculated into MacConkey agar (Oxoid, United Kingdom), chocolate agar, and mannitol salt agar. All cultures were incubated aerobically at 35–37°C for 24–48 hours. Routine laboratory bench procedures including morphological characterization and biochemical tests (coagulase test, catalase reaction, mannitol fermentation, and deoxyribonuclease (DNase test)) were subsequently performed. Identification of GNB was carried out via the use of colony morphology on MacConkey and biochemical tests including triple sugar iron (TSI), urease test, citrate tests, and sulfur indole and motility (SIM) tests.

#### 2.4.2. Drug Susceptibility Tests

Minimum inhibition concentrations (MICs) for a standard panel of antibiotics were evaluated on the basis of breakpoints set by the 2017 Clinical & Laboratory Standards Institute standards (CLSI M100-S25) [16]. In this process, test and control organisms were first suspended in normal saline to McFarland 0.5 standard. The suspensions were seeded onto Mueller–Hinton agar (MHA) (Oxoid, UK). The disks to be tested were subsequently placed onto the media and incubated at 37°C for 24 hrs. Antimicrobials tested against specific Gram-negative rods (GNRs) and other isolates included cefalexin; ceftriaxone 30 *μ*g; ceftazidime 30 *μ*g; tetracycline 30 *μ*g; gentamicin 10 *μ*g and amikacin-30 *μ*g; trimethoprim/sulfamethoxazole (TMP-SMX) (cotrimoxazole); chloramphenicol 30 *μ*g; ampicillin 10 *μ*g; nitrofurantoin 300 *μ*g; and ciprofloxacin 5 *μ*g.

Gram-positive isolates (mostly *Staphylococcus aureus* isolates) were tested for the following antimicrobials: vancomycin 30 *μ*g; chloramphenicol; penicillin 10 units; trimethoprim/sulfamethoxazole 1.25/23.75 *μ*g; ciprofloxacin 5 *μ*g; gentamicin 10 *μ*g; tetracycline class (tetracycline-30 *μ*g); nitrofurantoin 300 *μ*g; clindamycin 2 *μ*g; erythromycin 15 *μ*g; oxacillin 30 *μ*g; and rifampicin 5 *μ*g. The antimicrobial disks used for the stated tests were sourced from Oxoid Ltd., England. These drugs were selected based on the national list of medicines (Eritrean Formulary) to treat bacterial infections. Prescription frequencies and overall availability were also considered. Susceptibility testing was not performed for all Gram-positive isolates.

#### 2.4.3. Characterization of MDR, MRSA, and VRSA

Multidrug-resistant (MDR) was defined as nonsusceptibility to at least one agent in ≥3 antimicrobial drug classes [17]. Potential methicillin-resistant *Staphylococcus aureus* (MRSA) resistance was typed using disk diffusion (Oxoid™ disks). The double-disk diffusion test (D test) was also performed to evaluate clindamycin resistance when discrepant macrolide test results were obtained (e.g., erythromycin resistant and clindamycin susceptible). In this test, a 2 *μ*g clindamycin disk is placed in close proximity (20 mm apart) to a 15 *μ*g erythromycin disk and agar plate that has been inoculated with a staphylococcal isolate and incubated overnight. D-test-positive isolates will exhibit a flattening of the clindamycin zone adjacent to the erythromycin disk, thereby displaying the characteristic D-like pattern.

### 2.5. Quality Control

Reference strains including *S. aureus* (ATCC-25923), *E. coli* (ATCC-25922), and *P. aeruginosa* (ATCC-27853) were used to quality control microbiological procedures such as staining, biochemical identification procedures, and drug susceptibility testing.

### 2.6. Data Analysis

The data were analyzed using Statistical Package for Social Sciences (SPSS Inc., version 20.0; IBM, Chicago, IL, USA). Tables and figures were used to report the data. The 2015 Standards Institute (CLSI M100-S25) breakpoints were used for all antimicrobial agents. Test outcomes were reported either as susceptible (S) or intermediate (I) or resistant (R).

### 2.7. Ethical Considerations

Ethical approval for the study was obtained from the Eritrean Ministry of Health (MOH) research ethical committee. In addition, requisite permission was obtained from the hospital director and local administration. Participants were recruited voluntarily following provision of information on the study objective, study procedures, possible adverse effects, and the right to refuse or terminate their participation in the study at any time/stage. Information on the maintenance of data confidentiality and integrity was also provided. To ensure data confidentiality and privacy, personal identifiers such as names were not collected.

## 3. Results

### 3.1. General Characteristics of the Study Participants and Associated Surgical Procedures

This study enrolled 83 patients—25(30.1%) were females and 58 (69.9%) were males. The average age (±SD) of the patients was 42.65 ± 13.14 years (minimum 2 and maximum 82 years). Additional information on age grouping is shown in Table 1. In terms of institutional distribution, 48 (50.4%) of the patients were from the Halibet hospital and 35 (49.6%) were from the Orotta hospital. Amputations were the most common surgical procedure 35 (42.2%) followed by 19(22.9%) surgical debridement; 8(9.6%) fixation (e.g., open reduction and internal fixation (ORIF) and/or removal of internal fixation devices); and 14 (16.9%) colonoscopy and appendectomy. Additional surgical procedures contributed 8 (9.6%) of the reported proportions.

### 3.2. Bacteria Isolated

In this study, the most common isolates included *Citrobacter* spp. 15 (25%), *Klebsiella pneumoniae* 10 (15.6%), *Escherichia coli* 10 (15.6%), *Pseudomonas aeruginosa (8 (12.7%), Salmonella* spp*. 6(9.52%)*, *Enterobacter* spp. 5 (7.94), and *Acinetobacter* spp. 2 (3.17). In contrast, the most common Gram-positive bacteria isolates included *Staphylococcus aureus* 20 (40.8%), CONS (19 (38.78%), Gram-positive bacilli 7 (14.29%), and *Streptococcus viridans* 3 (6.12%) (Table 2). Overall, single bacterial isolates were recovered from 53 (63.9%) patients, while the rest 30 (36.1%) had polymicrobial infections.

### 3.3. Antimicrobial Resistance Pattern: Gram-Positive Isolates

**Figure 1:**
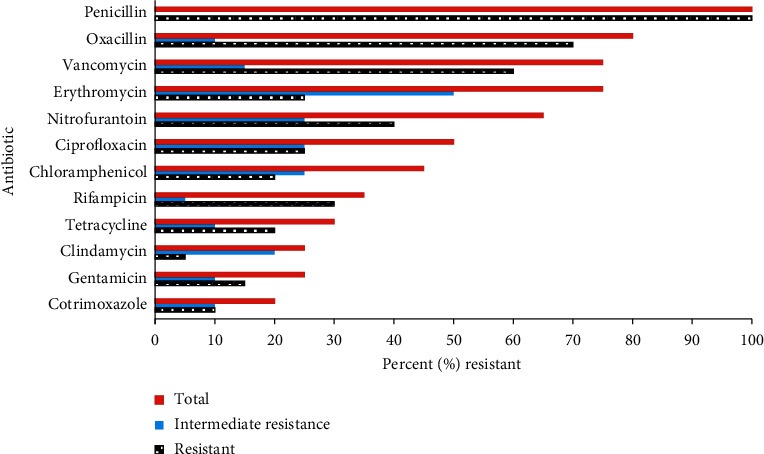
Disk diffusion test for Gram-positive isolates on 12 antibiotics, on Mueller–Hinton agar with 4% NaCl, as recommended in the Clinical and Laboratory Standards Institute guidelines.

The drug resistance profile of the 20 *Staphylococcus aureus* isolates was evaluated. In this analysis, 20 (100%) of the isolates were resistant to penicillin, 14 (70%) were resistant oxacillin, and 12 (60%) were resistant to vancomycin. Similarly, 5 (25%) were resistant to clindamycin. A similar proportion of isolates were resistant to Erythromycin (Figure 1). Resistance profiles for CONS, *Streptococcus viridans*, and Gram-positive bacilli were not evaluated.

### 3.4. Antimicrobial Susceptibility: Gram-Negative Isolates

The antimicrobial agents and number of proportions of isolates determined to be resistant and intermediate are presented in Table 3. The agent with the highest level of resistance was ampicillin with *Citrobacter* spp*., Escherichia coli, Klebsiella* spp*., Enterobacter* spp., and *Pseudomonas* spp. isolates exhibiting 100% resistance. The highest susceptibility rates recorded for Gram-negative bacteria (with the exception of *Klebsiella*) were to amikacin.

*Escherichia coli* isolates exhibited high resistance (>60% resistance) to chloramphenicol, ciprofloxacin, trimethoprim-sulfamethoxazole, gentamicin, nitrofurantoin, tetracycline, cefalexin, ceftazidime, and ceftriaxone. However, no resistance was observed for amikacin. Similarly, *Klebsiella* spp. isolates exhibited high resistance (>60%) to nitrofurantoin, cefalexin, ceftazidime, and ceftriaxone. High level of resistance (80% resistance) was also observed for *Pseudomonas* spp. isolates to nitrofurantoin, tetracyclines, cefalexin, ceftazidime, and ceftriaxone. *Citrobacter* spp. isolates had >70% resistance to several agents including tetracyclines, cefalexin, ceftazidime, and ceftriaxone. The two *Acinetobacter* isolates had 100% resistance to nitrofurantoin, tetracycline, cefalexin, ceftazidime, and ceftriaxone. See Figure 1 for additional resistance profiles.

### 3.5. Multidrug-Resistant (MDR) Patterns of Gram-Negative Isolates

The MDR patterns of specific isolates are presented in Table 4. In this analysis, 65 (97%) of the isolates were resistant to ≥3 classes of antimicrobial agents with varying combination of agents. In general, 8 (100%) of the *Pseudomonas aeruginosa* were resistant to >6 of the tested agents. Similarly, 10 (100%) of the *Escherichia coli* isolates were resistant to >5 agents. The two *Acinetobacter* isolates were resistant to >7 antimicrobial agents. We also noted that 4 (60%) of the *Klebsiella* isolates were resistant to >5 antimicrobial agents. The proportion of *Salmonella* isolates which were resistant to >6 antimicrobial agents was 67%.

### 3.6. Multidrug-Resistant (MDR) Patterns of *Staphylococcus aureus*

Analysis of the combinations of antimicrobial agent-specific isolates of *Staphylococcus aureus* that were susceptible are presented in Table 5. Results indicate that all the isolates were resistant to penicillin. At the same time, 17 (85%) of the isolates were resistant to >2 classes of antimicrobial agents. The proportion of isolates which were identified as MRSA was 14 (70). For MRSA, high sensitivity to clindamycin, erythromycin, trimethoprim-sulfamethoxazole, and gentamicin was observed. The proportion of MRSA which were resistant to vancomycin was 9 (64%).

## 4. Discussion

In hospital wards in SSA, the risk of nosocomial infections (SSIs, in particular) is often high [7, 9, 11, 12, 18, 19]. Multiple reports from the region have implicated a variety of GNB as the dominant agents. In this study, the most common isolates included *Citrobacter* spp. (25.4%), *Klebsiella* spp*. (15.87%), Escherichia coli (12.7%)*, *Proteus* spp. *(12.7%), Salmonella* spp. (12.7%), *Enterobacter* spp. (7.94%), and *Acinetobacter* spp*. (3.17%).* A significant number of *Pseudomonas aeruginosa* isolates were also present (12.7%). In contrast, Gram-positive bacteria isolates were predominantly *Staphylococcus aureus* (40.8%), CONS (38.78%), Gram-positive bacilli (14.29%), and *Streptococcus viridians* (6.12%). The dominance of these isolates in SSIs has been reported by multiple investigators in the region. The findings from this study support evidence that despite significant intra- and/or intercountry variation along with institutional variation, *Klebsiella* spp.*, E. coli*, *Proteus* spp., and *Pseudomonas aeruginosa* typically switch places between the first, second, third, and fourth most commonly isolated GNB in a majority of settings in SSA [4, 9, 18, 20].

The predominance of the relatively uncommon *Citrobacter* spp. in this setting is notable. However, high frequencies of *Citrobacter* spp. isolates have been reported in some studies from the region. For instance, a study conducted in an intensive care unit in Kenyatta National Hospital (one of the largest referral hospitals in East and Central Africa) reported high frequency of *P. aeruginosa, Klebsiella, Citrobacter* spp., and *S. aureus* [9]. The low frequency of *Acinetobacter* spp. isolates is also in harmony with some reports from the region [9]. In contrast, the dominant Gram-positive isolate was *Staphylococcus aureus*. Beyond the current results, the normal flora nature of some of these isolates on skin and mucosal surfaces along with the acquisition of diverse extraintestinal virulence factors and AMR mechanisms provides possible justification for their emergence as a threat to postoperative and ICU patients.

*In vitro* analysis of AMR in multiple GNBs noted a high level of resistance to commonly used antibiotics like ampicillin, trimethoprim-sulfamethoxazole, and tetracycline. Resistance of specific isolates to cefalexin, ceftriaxone, and ceftazidime was also high. To illustrate, the *E coli* isolated in this study were 100% resistant to ampicillin and >80% trimethoprim-sulfamethoxazole, tetracycline, cefalexin, ceftriaxone, and ceftazidime. High resistance (>80%) to multiple antibiotics (ampicillin, nitrofurantoin, and ceftriaxone) was also observed for *Klebsiella pneumoniae* isolates. This result is in harmony with some reports from the region [21]. Sensitivity of the *Klebsiella pneumoniae* isolates to amikacin and ciprofloxacin was also high. Furthermore, the isolated *Pseudomonas aeruginosa* were 100% resistant to nitrofurantoin, ampicillin, ceftriaxone, and ceftazidime. High level of resistance to tetracycline and trimethoprim-sulfamethoxazole was also noted in this isolates. Another atypical outcome observed in this study was the high resistance of most isolates to nitrofurantoin—an antibiotic indicated for urinary tract infections (UTIs) and for prophylactic use in recurrent UTI. An explanation for this outcome is unclear.

A number of issues are notable from these results. On the whole, the extensive postprocedural contamination of surgical sites by enterobacterial isolates is concerning. In particular, the high proportion of third-generation cephalosporin-resistant *Escherichia coli* (G3CREC) should raise concern, given its potential for pathogenicity [22]. This observation is consistent with previous studies in SSA, which have repeatedly noted that the frequency of G3CREC in the region is disproportionately high [23–25]. Emphasizing these concerns, a recent report noted that resistance to cephalosporins are increasing among nosocomial and community-acquired strains of GNBs worldwide [4] and that the high BoD associated with these strains (including G3CREC) will inevitably increase [22].

Multiple drivers of the projected increase exist. According to some reports, imprudent antibiotic use and poor adherence to infection-control practices are the keystone issues [26]. In SSA, these fundamental issues are poorly documented. In Eritrea for instance, there are no reports on the level of compliance by hospitals/healthcare workers to transmission-based control guidelines such as active surveillance to identify colonized patients and workers, decolonization of patients and healthcare workers, environmental decontamination or the widespread use of rub-in hand disinfection techniques. Research on the effectiveness of infection control practices (decontamination, disinfection, and sterilization) is particularly lacking. Another driver of the observed outcome is the extensive institutional use of cephalosporins in preoperative antibiotic prophylaxis (ceftriaxone and ampicillin are used extensively in this setting). This issue has been identified as a major concern in the region [4, 24, 25]. Indeed, it is our opinion that the widespread use of these medications in this setting should be re-evaluated.

In the subsequent analysis of multidrug resistance by GNBs, our investigation indicates that nearly all the isolates were resistant to >3 classes of antibiotics. Resistance or intermediate resistance (IR) to all the antibiotics tested in this study was observed in *E coli, Pseudomonas aeruginosa*, *Klebsiella pneumoniae*, and *Citrobacter* spp. isolates (Table 4). Frequently, ESBL-encoding plasmids contain linked resistance determinants (class A and AmpC *β*-lactamases (class C), among others) for the third-generation cephalosporins, SMX, tetracyclines, and aminoglycosides [10]. Resistance to this group of agents was noted in multiple isolates. In this regard, the result dovetails well with multiple reports which have noted the presence of extended and MDR isolates [23, 24]. The fact that resistance to the third-generation cephalosporins can be regarded as surrogate markers for MDR must also be noted [22].

Clearly, infection with MDR represents a formidable burden for both patients and healthcare systems. Importantly, the MDR strains and their intrinsic resistance to less costly antibiotics that are widely used in resource-limited settings like Eritrea confer limited treatment options for affected patients. Attention should also be directed at the high frequency of MDR *Pseudomonas aeruginosa* isolates. Many potential reservoirs of *Pseudomonas aeruginosa* have been identified in the hospital environment. These include cleaning solutions/disinfectants, sinks, respiratory equipment, drapes, endoscopes, physiotherapy pools, and vegetables among others. This catalogue of potential risk factors is often overlooked in infection control guidelines in SSA.

As previously described, analysis of the resistant profiles of the *Staphylococcus aureus* isolates was performed. Currently, pathogenic strains of *Staphylococcus aureus* are one of the most common pathogens in hospitals worldwide. The rapidly evolving pathogenicity of *Staphylococcus aureus* is associated with the capacity of this pathogen to produce several virulence factors including enterotoxin serotypes A through Q (SEA-SEQ), cytolytic toxins (*α*- and *β*-hemolysin), exfoliative toxins, toxic shock syndrome toxin-1 (TSST-1), Panton–Valentine leukocidin (PVL), protein A, and several enzymes [19]. Apart from ceftobiprole, the presence of modified penicillin-binding protein (PBP2′ or PBP2a) encoded by *Mec*A confers resistance to all *β* lactam antibiotics [19]. Remarkably, all the *Staphylococcus aureus* isolates in this study were resistant to penicillin. This phenotype has been described by several investigators in the region [13, 27–31]. The magnitude of HA-MRSA in our study was also high (70%). High prevalence of HA-MRSA has also been reported in the region [12–14, 31, 32]. Crucially, this finding is consistent with the findings of a recent study in the same institution (MRSA prevalence >72%) [14]. Therefore, the high frequency of MRSA reported in these studies appears to suggest that the crisis of MDR in Eritrea is a rapidly developing problem requiring urgent attention and resolution. A quandary in need of urgent determination is whether antimicrobial susceptibility patterns observed in this location are epidemiologically related to cases in other geographic locations in the country or region? To resolve this problem, molecular characterization of isolates from specific epidemiological clusters within the country should be a principle focus.

Finally, we have to reiterate the fact that although the level of resistance to clindamycin and erythromycin was low, a concerning level of resistance was observed. This concern is particularly reinforced by the consensus that inducible resistance to clindamycin exhibited by some strains of *Staphylococcus aureus* warrants cautious use of this agent in the treatment of erythromycin-resistant strains. Equally concerning is the high level of resistance to vancomycin (drug of choice for HA-MRSA). The high levels of vancomycin resistance reported in this study should be treated with some caution. A foremost concern is the fact that the procedures commonly used to detect vancomycin-intermediate *S. aureus* (VISA) strains or VRSA in the regions have some limitations. More importantly, detection of these isolates should be confirmed by a reference method—a process that was not performed in this study. Regardless, we have to highlight the fact that the finding corroborates previous reports [17] and aligns with several studies in the region [12–14]. In this regard, it reinforces our previous argument that VRSA is more prevalent in parts of East Africa (Ethiopia, Eritrea, Sudan) compared with southern parts of Africa [14, 33]. More importantly, these preliminary results highlight the need for the adoption of better testing procedures for VISA or VRSA in the country.

### 4.1. Strength and Limitations

To our knowledge, this is the first study, performed in an internationally accredited laboratory (with requisite internal quality and external quality assurance measures) to report on the phenotypic antibiotic resistance patterns of Gram-positive and Gram-negative isolates from SSIs in two of the largest publicly funded referral hospitals in Eritrea. The data may be important in designing interventions (surveillance programs included) for prevention of SSIs in healthcare facilities and for drawing up effective therapeutic guidelines. However, the study had several limitations including failure to perform anaerobic culture and molecular characterization of the isolates. Another notably finding which should be treated with a lot of caution is the high resistance to vancomycin. Regarding this concern, we have to emphasize the fact that the disk diffusion procedure used in this study or in most studies from the region has limitations in detecting resistance and that detection of these isolates should be confirmed by a reference method. In addition, sensitivity to specific drugs such as linezolid, piperacillin/tazobactam, and carbapenems (e.g., imipenem), among others, was not evaluated.

## 5. Conclusion

In this study, *Citrobacter* spp., *Klebsiella* spp*., Escherichia coli, Proteus* spp., *Pseudomonas aeruginosa*, *Salmonella* spp*., Enterobacter* spp*.,* and *Acinetobacter* spp. were the most common isolates. In contrast, the predominant Gram-positive isolates included *Staphylococcus aureus,* CONS, and *Streptococcus viridians*. Furthermore, it is clear that the significant exposure of patients to MDR including HA-MRSA and VRSA represents a significant problem for countries like Eritrea. Much work is needed in Eritrea to formulate stricter policies and guidelines on infection control practices (decontamination of surfaces, antimicrobial surveillance, antibiotic stewardship, cohorting and decolonization of patients and healthcare workers, etc.). Concomitant improvement and expansion/decentralization of diagnostic services and establishment of a central organizing body to routinely collate, analyze, and report relevant data should also be considered.

## Figures and Tables

**Table 1:** Characteristics of patients with positive culture from surgical wards in the Halibet and the Orotta National Referral Hospital in Asmara, Eritrea, 2017.

Variable	Type of surgery					Total *N* (%)
	Amputation *N* (%)	Col + Append *N* (%)	Debridement *N* (%)	Fixation *N* (%)	Others *N* (%)	
*Sex*						
Female	8 (32.0)	6 (24.0)	5 (20.0)	1 (4.0)	5 (20.0)	25 (30.1)
Male	26 (44.8)	8 (13.8)	14 (24.1)	7 (12.1)	3 (5.2)	58 (69.9)

*Occupation*						
Farmer	9 (47.4)	4 (21.1)	4 (21.1)	1 (5.3)	1 (5.3)	19 (22.9)
House wife	7 (43.8)	3 (18.8)	2 (12.5)	1 (6.2)	3 (18.8)	16 (19.3)
Self-employed	5 (55.6)	0 (0.0)	2 (22.2)	1 (11.1)	1 (11.1)	9 (10.8)
Student	1 (6.2)	3 (18.8)	8 (50.0)	2 (12.5)	2 (12.5)	16 (19.3)
Others	12 (52.2)	4 (17.4)	3 (13.0)	3 (13.0)	1 (6.2)	23 (27.7)

*Hospital*						
Halibet	19 (39.6)	2 (4.2)	15 (31.2)	8 (16.7)	4 (8.3)	48 (58.8)
Orotta	15 (42.9)	12 (34.3)	4 (11.4)	0 (0.0)	4 (11.4)	35 (42.2)

*Duration of operation*						
<60 minutes	17 (40.5)	7 (16.7)	8 (19.0)	4 (9.5)	6 (14.3)	42 (50.4)
>60 minutes	17 (41.5)	7 (17.1)	11 (26.8)	4 (9.8)	2 (4.9)	41 (49.6)

*Age of patient*						
<18 years	2 (12.5)	4 (25.0)	4 (25.0)	3 (18.8)	3 (18.8)	16 (19.3)
18–35 years	5 (27.8)	3 (16.7)	6 (33.3)	1 (5.6)	3 (16.7)	18 (20.7)
36–60 years	13 (50.0)	4 (15.4)	5 (19.2)	2 (7.7)	2 (7.7)	26 (31.3)
>60 years	14 (60.9)	3 (13.0)	4 (17.4)	2 (8.7)	0 (0.0)	23 (27.7)

Col + Append: colostomy and appendectomy.

**Table 2:** Distribution of bacterial isolates in relation to type of operation among patients in surgical wards at the Halibet and the Orotta National Referral Hospital in Asmara, Eritrea, 2017.

Variable	Type of surgery					Total *N* (%)
	Amputation	Col + Append	Debridement	Fixation	Others	
*Gram-positive bacteria*						
Gram-positive bacilli	4 (57.14)	1 (14.29)	1 (14.29)	1 (14.29)	0 (0.0)	7 (14.29)
CONS	7 (36.84)	3 (15.79)	4 (21.05)	4 (21.05)	1 (5.26)	19 (38.78)
*Staphylococcus aureus*	8 (40.0)	3 (15.0)	3 (15.0)	3 (15.0)	3 (15.0)	20 (40.8)
*Streptococcus viridians*	1 (33.33)	1 (33.33)	1 (33.33)	0 (0.0)	0 (0.0)	3 (6.12)
Total N (%)	20 (40.82)	8 (16.33)	9 (18.37)	8 (16.33)	4 (8.16)	49 (100)

*Gram-negative bacteria*						
*Citrobacter* spp.	7 (47.0)	3 (20.0)	2 (13.3)	1 (6.7)	2 (13.3)	15 (25.40)
*Proteus* spp.	3 (37.5)	0 (0.0)	3 (37.5)	0 (0.0)	2 (25)	8 (12.7)
*Escherichia coli*	3 (30.0)	3 (30)	3 (30)	0 (0.0)	1 (10)	10 (15.6)
*Klebsiella* spp.	2 (20.0)	1 (10.0)	5 (50)	2 (20.0)	0 (0.0)	10 (15.6)
*Acinetobacter* spp.	1 (50.0)	0 (0.0)	1 (50.0)	0 (0.0)	0 (0.0)	2 (3.17)
*Enterobacter* spp.	3 (60.0)	0 (0.0)	2 (40.0)	0 (0.0)	0 (0.0)	5 (7.94)
*P. aeruginosa*	5 (62.5)	2 (25.0)	1 (12.5)	0 (0.0)	0 (0.0)	8 (12.7)
*Salmonella* spp.	1 (16.7)	2 (33.33)	2 (33.33)	1 (16.7)	0 (0.0)	6 (9.52)
Total N (%)	25 (39.1)	11 (17.2)	19 (30.0)	4 (6.25)	5 (7.8)	64 (100)

CONS: coagulase-negative staphylococci; Col + Append: colostomy and appendectomy.

**Table 3:** Disk diffusion test for Gram-negative isolates on 11 antibiotics, on Mueller–Hinton agar with 4% NaCl, as recommended in the Clinical and Laboratory Standards Institute guidelines.

Bacteria isolated		Total no.	Antimicrobial agent tested										
			C *N* (%)	CIP *N* (%)	SXT *N* (%)	GN *N* (%)	F *N* (%)	TC *N* (%)	Amp. *N* (%)	CEP *N* (%)	CFT *N* (%)	CFR *N* (%)	Ami *N* (%)
Citrobacter spp.	R	15	6 (40)	7 (47)	9 (60)	10 (67)	12 (80)	11 (73)	15 (100)	14 (93)	14 (93)	11 (73)	3 (20)
	I		2 (13)	1 (	0 (0)	1	0 (0)	1		0 (0)	1	1	7

*Proteus* spp.	*R*	8	3 (38)	2 (25)	2 (33)	2 (25)	7 (88)	6 (75)	4 (50)	5 (63)	4 (50)	1 (13)	0 (0)
	I		0	0	0 (0)	0 (0)	0 (0)	0 (0)		0 (0)	0 (0)	0 (0)	3

*Escherichia coli*	R	10	6 (60)	6 (60)	9 (90)	7 (70)	7 (70)	9 (90)	10 (100)	8 (80)	9 (90)	8 (80)	0 (0)
	*I*		0 (0)	3	0 (0)	0 (0)	0 (0)	0 (0)		0 (0)		0 (0)	3

*Klebsiella* spp.	R	10	3 (30)	2 (20)	5 (50)	4 (40)	9 (90)	4 (40)	10 (100)	6 (60)	8 (80)	4 (40)	2 (20)
	*I*		0 (0)	0 (0)	1	1	0 (0)	2	0 (0)	0 (0)	0 (0)	0 (0)	3 (38)

*Acinetobacter* spp.	*R*	2	1 (50)	1 (50)	1 (50)	1 (50)	2 (100)	2 (100)	2 (100)	2 (100)	2 (100)	2 (100)	0 (0)
	*I*		1	0 (0)	0 (0)	1	0 (0)	0 (0)	0 (0)	0 (0)	0 (0)	0 (0)	0 (0)

*Enterobacter* spp.	R	5	1 (20)	0 (0)	2 (40)	1 (20)	4 (80)	0 (0)	5 (100)	3 (60)	4 (80)	0 (0)	1 (20)
	*I*		0 (0)	2	0 (0)	1	0 (0)	2		0 (0)	1	1	0 (0)

*Pseudomonas* spp.	R	8	4 (50)	2 (25)	5 (63)	1 (13)	8 (100)	7 (88)	8 (100)	8 (100)	8 (100)	8 (100)	2 (20)
	*I*		1	0 (0)	2	1	0 (0)	1	0 (0)	0 (0)	0 (0)	0 (0)	1

*Salmonella* spp.	*R*	6	4 (67)	0 (0)	2 (33)	2 (33)	5 (83)	2 (33)	4 (67)	5 (83)	3 (50)	2 (33)	0 (0)
	*I*		0 (0)	0 (0)	0 (0)	0 (0)	1	2		2	0 (0)	0 (0)	0 (0)

Total		**64**	**28 (44)**	**20 (31)**	**35 (54)**	**28 (43)**	**54 (85)**	**41 (64)**	**58 (91)**	**51 (80)**	**52 (80)**	**36 (55)**	**8 (12)**

C: chloramphenicol; CIP: ciprofloxacin; SXT: trimethoprim-sulfamethoxazole; GN: gentamicin; F: nitrofurantoin; TC: tetracycline; Amp: ampicillin; Cep: cefalexin; CFT: ceftazidime; CFR: ceftriaxone; Ami: amikacin.

**Table 4:** Multidrug resistance patterns of Gram-negative isolates among patients in surgical wards at the Halibet and the Orotta National Referral Hospital in Asmara, Eritrea, 2017.

Resistant patterns of antibacterial agents tested	No. of agents	*Klebsiella*spp., *N* (%)	*Pseudomonas* spp., *N* (%)	*Acinetobacter* spp., *N* (%)	*Enterobacter*spp., *N* (%)	*Citrobacter* spp., *N* (%)	*Proteus*spp., *N* (%)	*Salmonella* spp., *N* (%)	*Escherichia coli*, *N* (%)	Total no. (%)
C, CIP, SXT, GN, F, TC, AMP, CEP, CFR, CFT	10	—	—	—	—	4 (26.7)	—	—	3 (30)	6 (10.45)

C, CIP, SXT, F, TC, AMP, CEP, CFR, CFT, AMI	10	—	1 (12.5)	—	—	—	—	—	—	1 (1.49)

C, CIP, GN, F, TC, AMP, CEP, CFR, CFT	9	—	—	1 (50)	—	—	—	—	1 (10)	2 (2.99)

C, CIP, SXT, GN, F, TC, Amp, CEP, CFT	9	2 (20)	—	—	—	—	—	—	—	2 (2.99)

C, CIP, SXT, GN, F, TC, AMP, CFR, CFT	9	—	—	—	—	—	1 (12.5)	—	—	1 (1.49)

C, CIP, SXT, GN, TC, AMP, CEP, CFR, CFT	9	—	—	—	—	—	—	—	1 (10)	1 (1.49)

C, CIP, SXT, GN, F, TC, AMP, CEP, CFR	9	—	—	—	—	—	1 (12.5)	—	—	1 (1.49)

C, SXT, GN, F, TC, AMP, CEP, CFR, CFT	9	1 (10)	—	—	—	1 (6.7)	—	—	1 (10)	1 (1.49)

CIP, SXT, GN, F, TC, AMP, CEP, CFR, CFT	9	—	—	—	—	1 (6.7)	—	—	—	1 (1.49)

CIP, SXT, GN, TC, AMP, CEP, CFR, CFT, AMI	9	—	—	—	—	1 (6.7)	—	—	—	1 (1.49)

C, GN, F, TC, AMP, CEP, CFR, CFT	8	—	—	—	—	1 (6.7)	—	—	—	1 (1.49)

C, SXT, F, TC, AMP, CEP, CFR, CFT	8	—	2 (25)	—	—	—	—	—	—	2 (2.99)

C, F, TC, AMP, CEP, CFR, CFT,	7	—	1 (12.5)	—	—	—	—	—	—	1 (1.49)

C, GN, F, TC, AMP, CEP, CFR	7	—	—	—	—	—	—	1 (16.7)	—	1 (1.49)

C, SXT, GN, AMP, CEP, CFR, CFT	7	—	—	—	—	—	—	—	1 (10)	1 (1.49)

CIP, SXT, F, AMP, CEP, CFR, CFT	7	—	1 (12.5)	—	—	—	—	—	—	1 (1.49)

CIP, SXT, TC, AMP, CEP, CFR, CFT	7	—	—	—	—	1 (6.7)	—	—	—	1 (1.49)

F, TC, TC, AMP, CEP, CFR, CFT	7	—	—	—	—	—	—	—	1 (10)	1 (1.49)

GN, F, TC, AMP, CEP, CFR, CFT	7	—	1 (12.5)	—	—	1 (6.7)	—	—	—	2 (2.99)

SXT, F, TC, AMP, CEP, CFR, CFT	7	—	1 (12.5)	1 (50)	—	—	—	—	—	2 (2.99)

SXT, GN, F, AMP, CEP, CFR, CFT	7	1 (10)	—	—	—	1 (6.7)	—	—	—	1 (1.49)

C, F, AMP, CEP, CFR, CFT	6	—	—	—	—	—	—	1 (16.7)	—	1 (1.49)

C, SXT, F, TC, AMP, CFR	6	—	—	—	—	—	—	1 (16.7)	—	1 (1.49)

C, SXT, GN, AMP, CEP, CFR	6	—	—	—	1 (20)	—	—	1 (16.7)	—	2 (2.99)

F, TC, AMP, CEP, CFT, CFT	6	—	1 (12.5)	—	—	—	—	—	—	1 (1.49)

CIP, SXT, TC, AMP, CFR	5	—	—	—	—	—	—	—	2 (20)	1 (1.49)

F, AMP, CEP, CFR, CFT	5	1 (10)	—	—	—	—	—	—	—	1 (1.49)

F, TC, AMP, CEP, CFR	5	—	—	—	—	—	—	—	—	1 (1.49)

F, TC, AMP, CFT, AMI	5	—	—	—	—	1 (6.7)	—	—	—	1 (1.49)

GN, TC, AMP, CFR, AMI	5	1 (10)	—	—	—	—	—	—	—	1 (1.49)

C, F, T, AMP	4	—	—	—	—	—	1 (12.5)	—	—	1 (1.49)

F, AMP, CEP, CFR	4	1 (10)	—	—	2 (40)	2 (13.3)	—	—	—	4 (5.97)

F, AMP, CFR, AMI	4	—	—	—	1 (20)	—	—	—	—	1 (1.49)

F, AMP, CFR, CFT	4	1 (10)	—	—	—	—	—	—	—	1 (1.49)

F, T, AMP, CEP	4	—	—	—	—	—	—	—	—	1 (1.49)

CEP, AMP, CFR	3	—	—	—	—	—	1 (12.5)	—	—	1 (1.49)

F, AMP, CEP	3	1 (10)	—	—	—	1 (6.7)	—	—	—	1 (1.49)

F, CEP, CFR	3	—	—	—	—	—	1 (12.5)	2 (33.3)	—	2 (2.99)

F, TC, AMP	3	—	—	—	—	—	1 (12.5)	—	—	1 (1.49)

SXT, F, AMP,	3	1 (10)	—	—	1 (20)	—	—	—	—	2 (2.99)

SXT, TC, AMP	3	—	—	—	—	—	—	—	—	1 (1.49)

F, TC	2	—	—	—	—	—	2 (25)	—	—	2 (2.99)

Total		10	8	2	5	15	8	6	10	64

C: chloramphenicol (30 µg); CIP: ciprofloxacin (5 µg); SXT: trimethoprim-sulfamethoxazole; GN: gentamicin (30 µg); F: nitrofurantoin; TC: tetracycline; Amp: ampicillin; Cep: cefalexin; CFR: ceftazidime; CFT: ceftriaxone; Ami: amikacin. All the tests were conducted on Mueller–Hinton agar as specified by the Clinical and Laboratory Standards Institute guidelines.

**Table 5:** Drug resistance patterns of Gram-positive isolates among patients in surgical wards at the Halibet and the Orotta National Referral Hospital in Asmara, Eritrea, 2017.

Combination of drugs (%)	No. of agents	Total no. (%)
C, F, CLI, ERT, P, Van, RIF	7	1 (5)
CIP, GN, F, ERT, Oxa, P, Van	7	1 (5)
C, SXT, F, Oxa, P, Van	6	1 (5)
C, GN, ERT, Oxa, P	5	1 (5)
C, TC, ERT, Oxa, P	5	1 (5)
CIP, GN, F, TC, Oxa	5	1 (5)
ERT, Oxa, P, Van, RIF	5	1 (5)
F, TC, Oxa, P, Van	5	1 (5)
F, Oxa, P, Van	4	1 (5)
F, P, Van, RIF	4	1 (5)
F, TC, P, RIF	4	1 (5)
Oxa, P, Van, RIF	4	1 (5)
CIP, Oxa, P	3	1 (5)
Oxa, P, Van	3	2 (10)
Oxa, P, Van	2	1 (5)
P, RIF	2	1 (5.3)
P, Van	2	1 (5.3)
P	1	2 (10)
Total		**100**

CONS: coagulase-negative staphylococci; P: penicillin; C: chloramphenicol (30 µg); CIP: ciprofloxacin; SXT: trimethoprim-sulfamethoxazole; GN: gentamicin; F: nitrofurantoin; T: tetracycline; Cli: clindamycin; ERT: erythromycin; Oxa: oxacillin; Van: vancomycin; RIF: rifampicin. All the tests were conducted on Mueller–Hinton Agar as specified by the Clinical and Laboratory Standards Institute guidelines.

## Data Availability

All data sets used for this study are available from the corresponding author on reasonable request.

## Abbreviations

AST: Antimicrobial susceptibility testing
CLSI: Clinical and Laboratory Standards Institute
ESBL: Extended spectrum beta lactamases
GNB: Gram negative bacteria
G3CREC: Third-generation cephalosporin-resistant Escherichia coli
GNB: Gram-negative bacteria
MDR: Multidrug resistance
HA-MRSA: Hospital-acquired methicillin-resistant Staphylococcus aureus
SSI: Surgical site infection
VRSA: Vancomycin-resistant *Staphylococcus aureus*
TSST-1: Toxic shock syndrome toxin-1
PVL: Panton–Valentine.
